# Combination of proteasome and HDAC inhibitor enhances HPV16 E7-specific CD8^+^ T cell immune response and antitumor effects in a preclinical cervical cancer model

**DOI:** 10.1186/s12929-014-0111-1

**Published:** 2015-01-16

**Authors:** Zhuomin Huang, Shiwen Peng, Jayne Knoff, Sung Yong Lee, Benjamin Yang, Tzyy-Choou Wu, Chien-Fu Hung

**Affiliations:** Department of Pathology, Johns Hopkins Medical Institutions, CRB II Room 307, 1550 Orleans Street, 21231 Baltimore, MD USA; Obstetrics and Gynecology, Johns Hopkins Medical Institutions, Baltimore, MD USA; Molecular Microbiology and Immunology, Johns Hopkins Medical Institutions, Baltimore, MD USA; Oncology, Johns Hopkins Medical Institutions, Baltimore, MD USA; Department of Gynecology, Shenzhen Maternity and Child Healthcare Hospital, Southern Medical University, Shenzhen, China; Department of Internal Medicine, Korea University Medical Center, Seoul, South Korea

**Keywords:** Bortezomib, SAHA, Vorinostat, Antitumor, Host immunity

## Abstract

**Background:**

Bortezomib, a proteasome inhibitor and suberoylanilide hydroxamic acid (SAHA, also known as Vorinostat), a histone deacetylase inhibitor, have been recognized as potent chemotherapeutic drugs. Bortezomib and SAHA are FDA-approved for the treatment of cutaneous T cell lymphoma and multiple myeloma/mantle cell lymphoma, respectively. Furthermore, the combination of the bortezomib and SAHA has been tested in a variety of preclinical models and in clinical trials and may be ideal for the treatment of cancer. However, it remains unclear how this treatment strategy affects the host immune response against tumors.

**Results:**

Here, we used a well-defined E6/E7-expressing tumor model to examine how the immune system can be motivated to act against tumor cells expressing tumor antigens. We demonstrate that the combination of bortezomib and SAHA elicits potent antitumor effects in TC-1 tumor-bearing mice. Additionally, we are the first to show that treatment with bortezomib and SAHA leads to tumor-specific immunity by rendering tumor cells more susceptible to killing by antigen-specific CD8+ T cells than treatment with either drug alone.

**Conclusions:**

The current study serves an important foundation for the future clinical application of both drugs for the treatment of cervical cancer.

**Electronic supplementary material:**

The online version of this article (doi:10.1186/s12929-014-0111-1) contains supplementary material, which is available to authorized users.

## Background

Bortezomib is a proteasome inhibitor recognized as a potent chemotherapeutic agent that is currently used to treat relapsed multiple myeloma and mantle cell lymphoma (for review see [1]). Bortezomib inhibits the 26S proteasome, which is a key regulator of intracellular protein degradation. The downstream effects of bortezomib include antitumor effects, which are the result of inhibiting tumor cell proliferation or promoting tumor cell apoptosis [2]. Bortezomib-induced tumor cell apoptosis may enhance the immunogenicity of tumor cells and provide an opportunity for generating tumor-specific immunity [3]. We have previously elucidated the immune mechanism of the antitumor effects of bortezomib in an ovarian cancer model and found that bortezomib can be used to promote the generation of antigen-specific CD8+ T cells [4]. It is important to further elucidate this mechanism, to determine the utility of bortezomib as a therapeutic agent in additional cancer models, and to identify other therapeutic agents that may enhance the antitumor effects of bortezomib.

Histone deacetylase inhibitors (HDACi) have been identified as a class of drugs with anticancer properties that can potentially be used in conjunction with bortezomib to further enhance its cancer therapeutic effects. HDACi inhibit an enzyme responsible for the deacetylation of histones, and lead to the expression of suppressed genes and regulation of abnormal cell growth [5]. HDACi may also contribute to cancer control by histone-independent mechanisms by modifying the acetylation of non-histone proteins such as p53 [6] and heat shock protein-90 [7]. These mechanisms produce antitumor effects including induced differentiation, cell growth arrest, and an increase in apoptosis [8,9]. Furthermore, it has been shown that treating cells with HDACi can lead to the upregulation of MHC class I and II molecules [10]. This suggests that tumor cells may become more susceptible to tumor-specific CD8+ T cell immunotherapy following treatment with an HDACi. One HDACi with therapeutic characteristics is suberoylanilide hydroxamic acid (SAHA, also known as Vorinostat), which is an FDA approved HDACi used in the treatment of cutaneous T-cell lymphoma [11]. SAHA may be an ideal drug to use in combination with bortezomib for the treatment of cancer.

Indeed, bortezomib and SAHA have previously been used in combination in a xenogeneic model for the treatment of cervical cancer [12]. This study found that bortezomib-mediated cervical cancer cell apoptosis might be facilitated by blocking E6-mediated proteasomal degradation of p53. Furthermore, because HPV oncoprotein E7 is known to interact with class I HDACs [13], Lin et al examined the effects of SAHA with bortezomib on tumor cell apoptosis. They found that the combination elicited synergistic killing of HPV-expressing cervical cancer cell lines and that the combination treatment diminished tumor growth of HeLa xenografts more effectively than either drug alone. Encouraged by these results, we took the opportunity to examine a different aspect of the effects of bortezomib and SAHA, namely, the host immune response against tumor cells.

In the current study, we used a well-defined E6/E7-expressing tumor model to examine how the immune system can be motivated to act against tumor cells expressing tumor antigens. We demonstrate that the combination of bortezomib and SAHA elicits potent antitumor effects in TC-1 tumor-bearing mice. Furthermore, we are the first to show that treatment with bortezomib and SAHA leads to tumor-specific immunity by rendering tumor cells more susceptible to killing by antigen-specific CD8+ T cells than treatment with either drug alone. Considering that both bortezomib and SAHA are FDA-approved for the treatment of specific types of cancer, this study has significant translational value.

## Methods

### Mice

Six- to 8-week-old female C57BL/6 mice were purchased from the National Cancer Institute (Frederick, MD, USA) and housed in the oncology animal facility of the Johns Hopkins Hospital (Baltimore, MD, USA). All animal procedures were performed according to approved protocols and in accordance with recommendations for the proper use and care of laboratory animals.

### Reagents and cell lines

We have previously generated an E7-expressing tumorigenic cell line, TC-1 [14], and a firefly luciferase-expressing TC-1 cell line, TC-1-luc [15]. The H-2Db-restricted HPV16 E7aa49-57 peptide, RAHYNIVTF, was synthesized by Macromolecular Resources (Denver, CO) at a purity of ≥80%. PerCP-Cy5.5-conjugated anti-mouse CD3 (clone 17A2), anti-mouse CD45 (clone 30-F11), FITC and PE-conjugated anti-mouse CD8a (clone 53.6.7), FITC-conjugated rat anti-mouse CD4 (clone RM4-5), FITC-conjugated rat anti-mouse IFN-γ (clone XMG1.2) antibodies were purchased from BD Pharmingen (San Diego, CA). PE-conjugated, HPV16 E749-57 peptide loaded H-2D^b^ tetramer was provided by NIAID tetramer core facility (Atlanta, GA). Commercially available bortezomib (PS341; Millennium Pharmaceuticals) was reconstituted according to the manufacturer’s instructions and diluted in 0.9% saline before *in vivo* administration. Suberoylanilide hydroxamic acid (SAHA, LC Laboratories) was dissolved in DMSO and then diluted in 2-Hydroxypropyl-β-cyclodextrin solution before each injection.

### Cell viability assay

To determine the viability of TC-1 cells after bortezomib and SAHA treatment, 3-(4,5-dimethyl-2-yl)-5-(3-carboxymethoxyphenyl)-2-(4-sulfophenyl)-2H-tetrazolium, inner salt (MTS, Promega) assay was performed. Briefly, TC-1 cells were plated in 96-well plates at a density of 1 × 10^3^ cells/well and incubated at 37°C in the presence of 5% CO_2_ for 12 hours. The cells were then treated with various concentrations of bortezomib or SAHA for 48 hours, respectively. At the end of the treatment period, MTS reagent was added to each well, and the plate was incubated for 4 hours at 37°C in the dark. After incubation, the absorbance was measured at 490 nm using the VERSA Max Microplate Reader. Data from three independent experiments were analyzed and normalized to the absorbance of wells containing media only (0%) and untreated cells (100%). The IC50 values were calculated from sigmoidal dose-response curves using MS Excel software. As shown in Additional file 1: Figure S1, the IC50 for bortezomib in TC-1 cells is 7.1 nM and that for SAHA is 25.7 μM.

### In vivo treatment experiments

C57BL/6 mice were inoculated subcutaneously with 3 × 10^4^ TC-1 cells/per mouse on day 0. The tumor-bearing mice were divided into four groups (5 per group) based on the treatment regimens: control (2-Hydroxypropyl-β-cyclodextrin solution only), bortezomib only, SAHA only, both bortezomib and SAHA. For the administration of bortezomib, 1 mg/kg of bortezomib was injected intraperitoneally on days 5, 8, 11, and 14 after tumor inoculation. For the SAHA administration, 30 mg/kg of SAHA was injected inraperitoneally into tumor-bearing mice daily from day 5 to day 14 after tumor inoculation. The control group received the vehicle alone using the same schedule as SAHA treatment.

### Tumor measurement

Tumor size was monitored by measuring the longest dimension (length) and shortest dimension (width) using dial calipers at 3-day intervals. Tumor volume was calculated by the following formula: tumor diameter = 0.5 × (length + width).

### Preparation of single-cell suspensions from TC-1 tumors

Four days after the last treatment, TC-1 tumors were resected from mouse, placed in RPMI-1640 medium containing 100U/ml penicillin and 100 μg/ml streptomycin and washed with PBS. The solid tumors were then minced into 1- to 2-mm pieces and immersed in serum-free RPMI-1640 medium containing 0.05 mg/ml collagenase I, 0.05 mg/ml collagenase IV, 0.025 mg/ml hyaluronidase IV, 0.25 mg/ml DNase I, 100 U/ml penicillin, and 100 μg/ml streptomycin and incubated at 37°C with periodic agitation. The tumor digest was then filtered through a 70-μm nylon filter mesh to remove undigested tissue fragments. The resultant single tumor cell suspensions were washed twice in Hank’s buffered salt solution (HBSS) (400 *g* for 10 min), and viable cells were determined using trypan blue dye exclusion.

### HPV16 E7-specific CD8^+^ T cell responses in tumor-bearing mice treated with bortezomib and/or SAHA

Groups of C57BL/6 mice (5 per group) were challenged with TC-1 tumor cells and treated with bortezomib and/or SAHA as described above. To detect HPV16 E7-specific CD8^+^ T cells in peripheral blood, peripheral blood mononuclear cells (PBMCs) were harvested from the tail vein one week after the last treatment. The cells were stained with FITC-conjugated anti-mouse CD8a (BD Pharmingen, San Diego, CA, USA) and PE-conjugated HPV16 E7 aa49-57 peptide loaded H-2D^b^ tetramer and acquired with FACSCalibur.

To detect HPV16 E7-specific CD8^+^ T cells in the tumor, single cell suspensions were stimulated with HPV16 E7 aa49-57 peptide (1 μg/ml) in the presence of GolgiPlug (BD Pharmingen, San Diego, CA, USA) overnight at 37°C. The cells were then stained with PE-conjugated anti-mouse CD8a. After permeabilization and fixation, the cells were stained with FITC-conjugated anti-mouse IFN-γ followed by flow cytometry analysis. The data were analyzed with FlowJo or CellQuest Pro software.

### IFN-γ secretion in E7-specific cytotoxic T cells induced by bortezomib and/or SAHA pretreated TC-1 cells

2 × 10^5^ TC-1 cells per well were plated in 6-well plates and treated with bortezomib (3.5 nM) and/or SAHA (12.5 μM) for 24 hours. The cells were then harvested and 1 × 10^5^ tumor cells per well were co-incubated with E7-specific cytotoxic T cells in 96-well plates at 37°C for 4 hours (E:T ratio of 1:1) at the presence of GolgiPlug. The cells were then harvested and stained with PE-conjugated CD8 and FITC-conjugated IFN-γ. The cells were acquired with FACSCalibur and analyzed with FlowJo.

### *In vitro* cytotoxic T cell assay

For the in vitro cytotoxic T cell assay, luciferase-expressing TC-1 tumor cells were added to 96-well plates (1 × 10^4^ per well) and incubated overnight at 37°C. The cells were then treated with bortezomib (3.5 nM) and/or SAHA (12.5 μM) for 24 hours and were used as target cells. After washing with PBS, HPV16 E7-specific cytotoxic T cells (CTLs), generated as previously described [16], were added to the target cells t different E:T ratios and incubated at 37°C for 4 hours. The target cells incubated without CTLs served as a negative control. Luciferin (1.3 × 10^−4^ mg per well) was added to the wells for optical imaging. The expression of luciferase was measured using the IVIS luminescence imaging system series 2000. Bioluminescence signals were acquired for 30 seconds.

### Statistical analysis

All experiments were replicated twice independently. All data were expressed as means ± standard error (SE) and are representative of at least two independent experiments. Otherwise indicated, the statistical significance of difference was assessed by two-tailed Student’s *t* test using SPSS version 16.0. The level of significance was set at *p* < 0.05.

## Results

### Combination treatment of bortezomib and SAHA generates potent antitumor effects in TC-1 tumor-bearing mice

**Figure 1:**
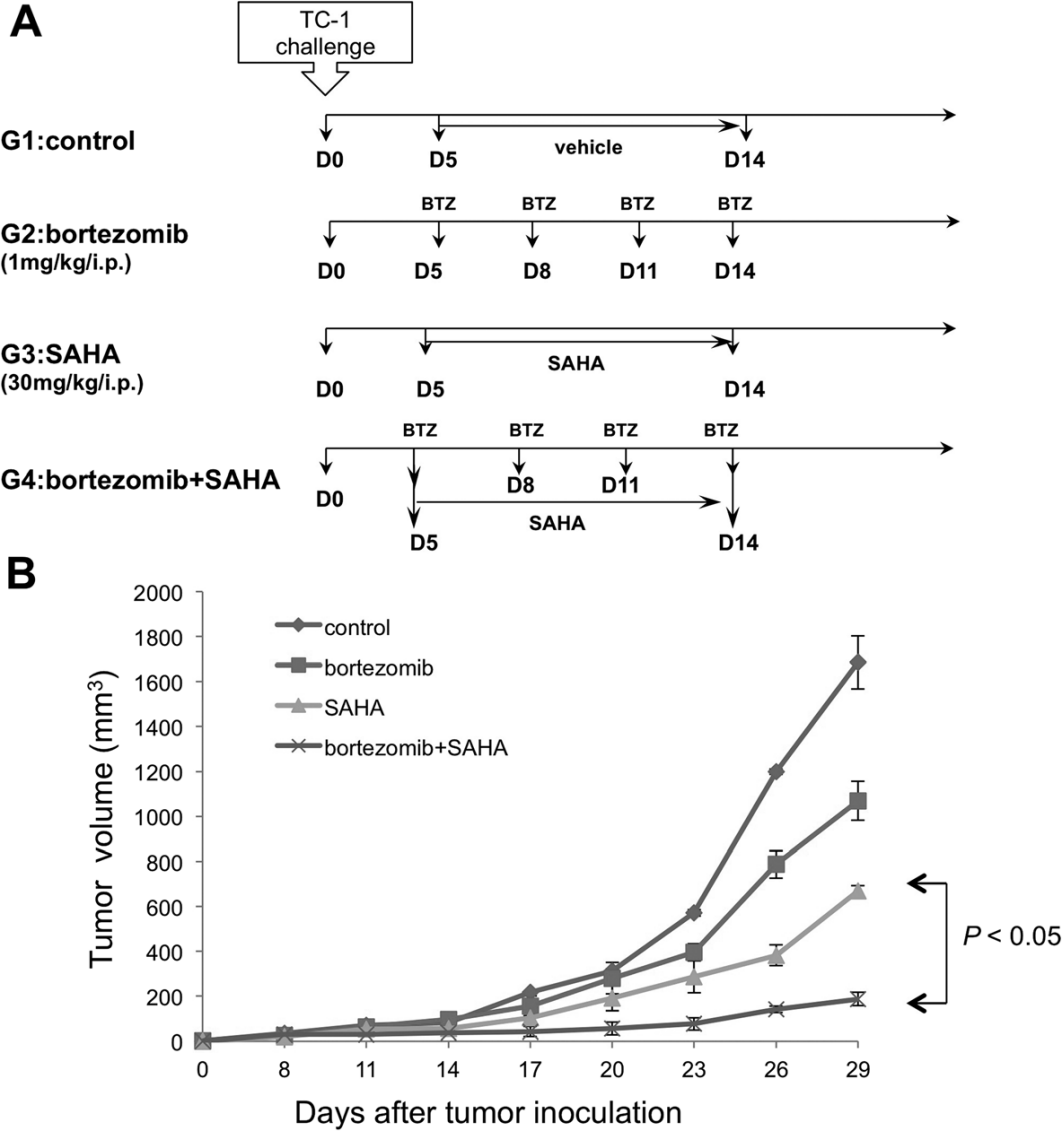
***In vivo*** **tumor treatment experiments.** Groups of C57BL/6 mice were subcutaneously challenged with 3 × 10^4^ TC-1 tumor cells per mouse on day 0. On day 5, the tumor-bearing mice were randomly divided into four groups (5 per group) and treated with one of the following regimens: vehicle, bortezomib alone (1 mg/kg, once every 3 days), SAHA alone (30 mg/kg, once per day), or the combination of bortezomib and SAHA. Bortezomib and SAHA were injected intraperitoneally and the treatment lasted for a total of 10 days. **A**. Schematic diagram of the treatment regimens. **B**. Line graph depicting the tumor volume in TC-1 tumor-bearing mice treated with bortezomib and/or SAHA (mean + SE; *p* < 0.05).

We first characterized the antitumor effects of the proteasome inhibitor bortezomib and the histone deacetylase inhibitor SAHA, alone or in combination against the E7-epressing TC-1 tumor model in C57BL/6 mice. Groups of mice were treated according to the regimens outlined in Figure 1A. Mice were challenged with TC-1 cells and then began the various treatments five days later. Bortezomib was injected intraperitoneally at 3 day intervals. SAHA was administered daily from day 5 to day 14 by intraperitoneal injection. As shown in Figure 1B and Additional file 2: Figure S2, mice treated with bortezomib or SAHA alone had lower tumor volumes and weights than mice treated with vehicle alone. Furthermore, mice treated with the combination of bortezomib and SAHA experienced significantly lower tumor volume than any other treatment regimen. These data suggest that bortezomib and SAHA elicit synergistic antitumor effects in TC-1 tumor-bearing mice.

### Combination treatment of bortezomib and SAHA elicits antigen-specific CD8+ T cells in TC-1 tumor-bearing mice

**Figure 2:**
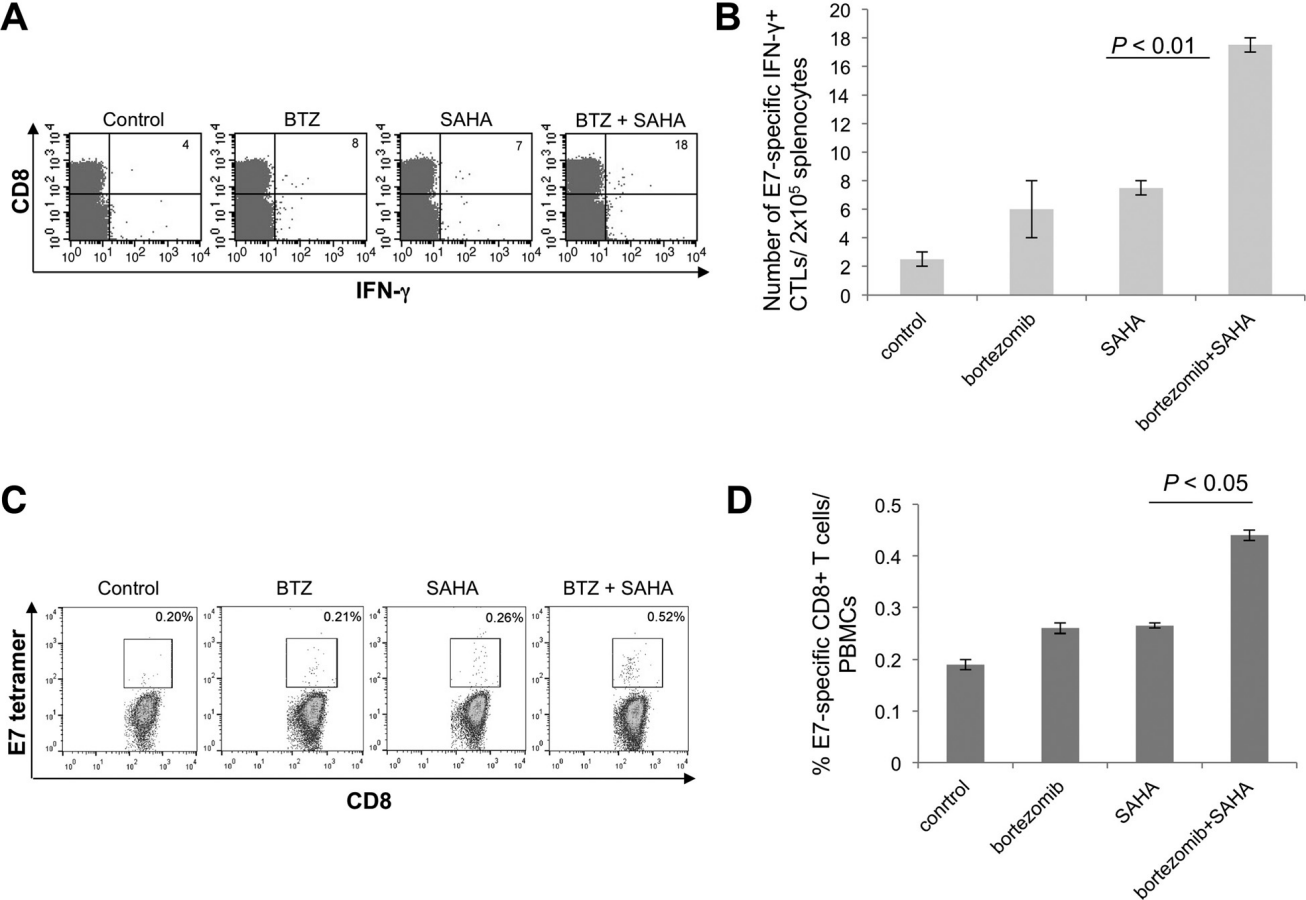
**Flow cytometry analysis to determine HPV16 E7-specific CD8** ^**+**^ **T cell responses in tumor-bearing mice treated with bortezomib and/or SAHA.** Groups of C57BL/6 mice (5 per group) were challenged with TC-1 tumor cells and treated with bortezomib and/or SAHA according to Figure 1A. Splenocytes from tumor-bearing mice were harvested one week after the last treatment and were stimulated with HPV16 E7aa49-57 peptide (1 μg/ml). The cells were then stained with PE-conjugated anti-mouse CD8a and FITC-conjugated anti-mouse IFN-γ and analyzed by flow cytometry. **A**. Bar graph depicting the number of E7-specific IFN-γ-secreting CD8+ T cells per 2 × 10^5^ pooled splenocytes (mean + SE; *p* < 0.01). **B**. Representative data of flow cytometry E7 peptide-loaded H-2D^b^ tetramer staining in the various groups. **C**. Peripheral blood mononuclear cells (PBMCs) were harvested from the tail vein one week after the last treatment. Cells were stained with FITC-conjugated anti-mouse CD8a and PE-conjugated HPV16 E7aa49-57 peptide loaded H-2D^b^ tetramer and acquired with FACSCalibur. **D**. Bar graph depicting the percentage of E7 tetramer CD8+ T cells in PBMCs (*p* < 0.05).

In order to characterize the antigen-specific cell-mediated immune responses generated by bortezomib and SAHA, groups of TC-1 tumor-bearing mice were treated as illustrated in Figure 1A. Splenocytes were harvested from tumor-bearing mice one week after the last treatment and stimulated with HPV16 E7 peptide (aa49-57). The splenocytes were stained for CD8 and IFN-γ expression and analyzed by flow cytometry. As shown in Figure 2A and B, a higher number of activated CD8+ T cells were among splenocytes isolated from mice treated with bortezomib and SAHA compared to those from mice treated with bortezomib or SAHA alone. Additionally, peripheral blood mononuclear cells (PBMCs) were harvested from the tail vein one week after the last treatment and then stained for CD8 expression and E7-specificity using E7 peptide-loaded H-2D^b^ tetramer. Mice treated with the combination of bortezomib and SAHA generated a significantly higher percentage of E7-specific CD8+ T cells among PBMCs compared to those treated with bortezomib or SAHA alone (Figure 2C and D). These data suggest that, together, bortezomib and SAHA have a better ability to generate antigen-specific CD8+ T cells than separately.

### Combination treatment of bortezomib and SAHA generates higher percentage of CD8+ T cells and antigen-specific CD8+ T cells in the tumor

**Figure 3:**
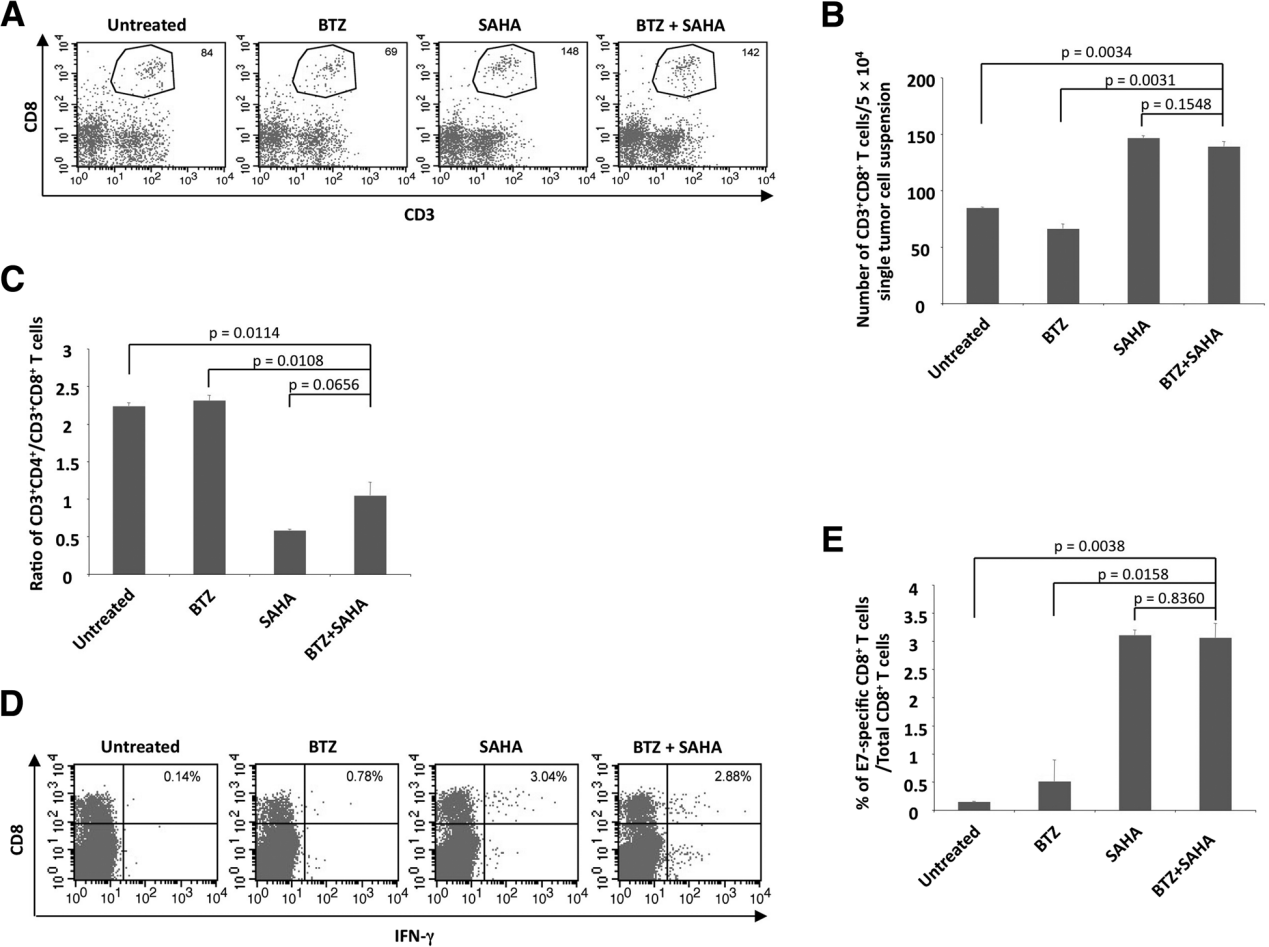
**Analysis of TC-1 tumor infiltrating lymphocytes after treatment.** Groups of female C57BL/6 mice (5 per group) were injected with 3 × 10^4^ of TC-1 tumor cells subcutaneously. Five days after tumor cell injection, the mice were treated through intraperitoneal injection with one of the following regimens: vehicle, bortezomib alone (1 mg/kg, once every 3 days), SAHA alone (30 mg/kg, once per day), or the combination of bortezomib and SAHA as described in Figure 1A. 4 days after last treatment, the tumors were resected from the mice and the single tumor cell suspension cells were prepared by enzymatic digestion. The cells were then stained with anti-mouse CD3, CD4 and CD8, or stimulated with HPV16 E7aa49-57 peptide at the presence of GolgiPlug overnight followed by flow cytometry analysis **A**. Representative of CD3 and CD8 staining images of TC-1 tumor single cell suspension. **B**. Summary of the infiltrating CD3^+^CD8^+^ T cells in TC-1 tumor. **C**. Summary of CD3^+^CD4^+^/CD3^+^CD8^+^ ratio of infiltrating lymphocytes in TC-1 tumor. **D**. Representative of IFN-γ intracellular staining images of TC-1 tumor infiltrating CD8+ T cells. **E**. Summary of IFN-γ intracellular staining data of TC-1 tumor infiltrating CD8+ T cells.

Next, we set out to characterize the immune responses generated by bortezomib and SAHA in the tumor microenvironment. TC-1 tumor-bearing mice were treated following the regimen described in Figure 1A. 4 days after the last treatment, tumors were resected and prepared into single tumor cells suspension by enzymatic digestion for analyses. As shown in Figure 3A and B, treatments with SAHA alone or SAHA in combination with bortezomib induce higher numbers of CD8+ T cells in the tumor. Furthermore, the ratios of CD8:CD4 T cells are also higher in the tumors of mice treated with SAHA alone or SAHA with bortezomib (Figure 3C). In addition, treatments with SAHA alone or SAHA combined with bortezomib result in the highest number of E7-specific IFN-γ + CD8+ T cells in the tumors (Figure 3D and F). These data show that treatments with SAHA alone or SAHA in combination with bortezomib generate higher percentage of CD8+ T cells and higher number of antigen-specific CD8+ T cells in the tumors. Importantly, although SAHA alone appears to induce CD8+ T cells immune responses in the tumors, only treatment with SAHA in combination with bortezomib results in the best antitumor effects (Figure 1B).

### Tumor cells treated with bortezomib and SAHA elicit potent antigen-specific CD8+ T cell immune responses

**Figure 4:**
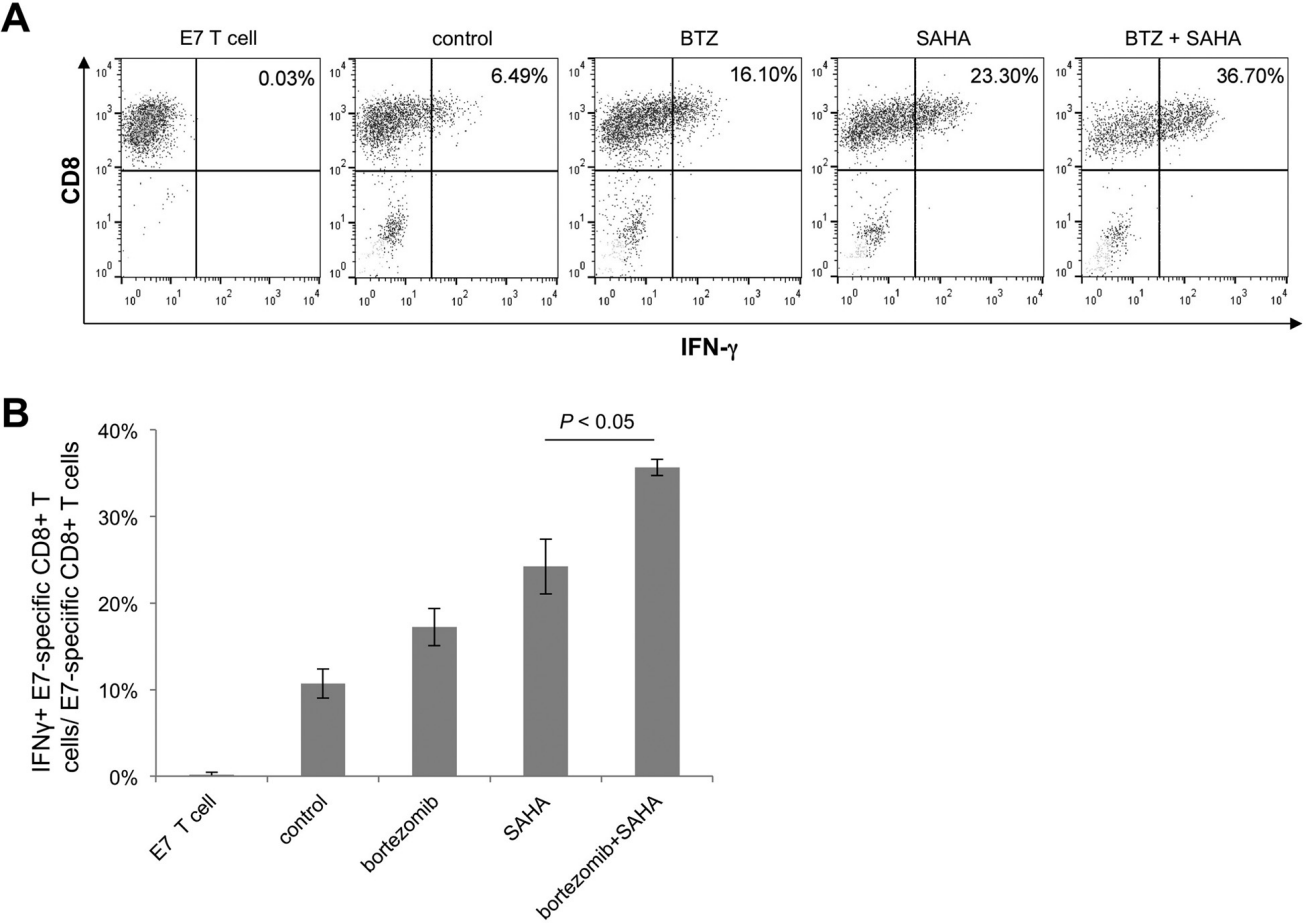
**Flow cytometry analysis to determine the ability of bortezomib and/or SAHA pretreated TC-1 cells to induce IFN-γ** **secretion in E7-specific cytotoxic T cells.** TC-1 cells were plated in 6-well plates (2 × 10^5^ /well) and treated with bortezomib (3.5 nM) and/or SAHA (12.5 μM) for 24 h. The cells were then harvested and 1 × 10^5^ tumor cells/well were co-incubated with E7-specific CD8+ T cells in 96-well for 4 h (E:T ratio of 1:1) in the presence of GolgiPlug. The cells were then harvested and stained with PE-conjugated CD8 and FITC-conjugated IFN-γ. Cells were acquired with FACSCalibur and analyzed with FlowJo. **A**. Representative flow cytometry data demonstrating the percentage of IFN-γ positive cells among E7-specific CTLs induced by TC-1 cells treated with bortezomib and/or SAHA. **B**. Bar graph depicting the percentage of IFN-γ + E7-specific CTLs among all E7-specific CTLs induced by TC-1 cells treated with bortezomib and/or SAHA.

We then treated TC-1 cells with bortezomib and/or SAHA and subsequently incubated them with E7-specific CD8+ T cells. Following incubation, cells were stained for CD8 and IFN-γ expression and analyzed by flow cytometry. Figure 4A and B show that a significantly higher percentage of E7-specific CD8+ T cells incubated with TC-1 cells treated with the combination of bortezomib and SAHA were activated, compared to those incubated with TC-1 cells treated with either bortezomib or SAHA alone. These data suggest that tumor cells treated with the combination of bortezomib and SAHA can activate potent E7-specific CD8+ T cell immune responses.

### TC-1 cells treated with the combination of bortezomib and SAHA are rendered susceptible to CD8+ T cell-mediated killing

**Figure 5:**
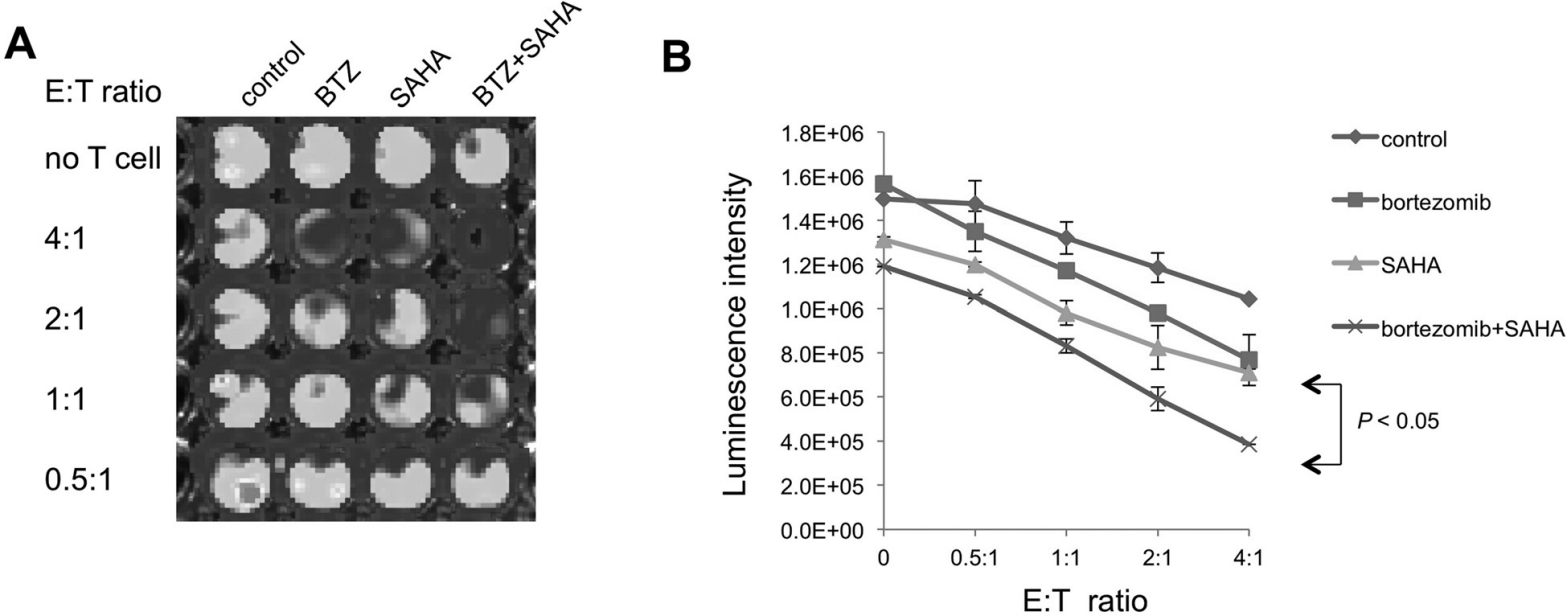
***In vitro*** **cytotoxic T cell assay.** Luciferase-expressing TC-1 tumor cells were added to 96-well plates (1 × 10^4^ per well) and incubated for 18 h at 37°C. The cells were then treated with bortezomib (3.5 nM) and/or SAHA (12.5 μM) for 24 h. After washing with PBS, HPV16 E7-specific CTLs were added to the cells and incubated at 37°C for 4 hours. The expression of luciferase was measured using the IVIS luminescence imaging system series 2000. Bioluminescence signals were acquired for 30 s. **A**. Representative luminescence images of 96-well plates showing tumor cell lysis. **B**. Line graph depicting the quantification of luminescence intensity in tumor cells treated with drugs and/or E7-specific CTLs in different E:T ratio data shown as mean ± SE.

In order to further elucidate the observed antitumor effects generated by bortezomib and SAHA in tumor-bearing mice (Figure 1), we examined the effect of bortezomib and SAHA treatment on the susceptibility of TC-1 cells to CD8+ T cell-mediated killing. Luciferase-expressing TC-1 cells were treated with bortezomib and/or SAHA and then incubated with E7-specific CD8+ T cells. As shown in Figure 5A and B, E7-specific CD8+ T cells elicited the most potent cytotoxic effect in the presence of TC-1 cells treated with both bortezomib and SAHA as demonstrated by decreased luminescence intensity. These data suggest that bortezomib and SAHA render TC-1 cells the most susceptible to E7-specific CD8+ T cell-mediated killing.

## Discussion

In the current study, we examined the effects of bortezomib and SAHA combination treatment on host immune responses against the TC-1 tumor model. We found that while bortezomib and SAHA elicited antitumor effects in TC-1 tumor-bearing mice, the combination of the two drugs created a synergistic effect. Furthermore, we demonstrated that the combination of bortezomib and SAHA generated significantly greater E7-specific CD8+ T cells in the spleen and circulation compared to treatment with either drug alone. We also showed that treatments with SAHA alone or SAHA in combination with bortezomib induce higher percentage of CD8+ T cells and more antigen-specific CD8+ T cells in the tumors, however, only the combination treatment resulted in the best antitumor outcomes. In addition, we showed that treatment of TC-1 cells with bortezomib and SAHA led to a significant increase in the potency of antigen-specific CD8+ T cell immune activation. Importantly, we demonstrated that the combination of bortezomib and SAHA rendered TC-1 cells the most susceptible to E7-specific CD8+ T cell-mediated killing, compared to either drug alone.

In the current study, we have observed a significant therapeutic anticancer effect when TC-1 tumor-bearing mice were treated with the combination of bortezomib and SAHA. Our results are consistent with previously reports of synergistic anticancer effects from the combination treatment with bortezomib and SAHA in other cancer models, such as prostate cancer [17], glioblastoma [18], multiple myeloma [19], and T-cell lymphoma [20]. Furthermore, several clinical trials combining bortezomib and SAHA have been reported in multiple myeloma [21], non small-cell lung cancer [22], children with refractory or recurrent solid tumors [23], and in patients with advanced solid tumors [24,25]. Most of these studies mainly focus on the therapeutic effect of the combination treatment rather than how it affects the host immune response and how it renders tumor susceptible to immune-mediated killing. Our results in cervical cancer are an important addition to this body of literature.

Here we show that treatments with either SAHA alone or SAHA in combination with bortezomib can induce higher percentage of CD8+ T cells and more antigen-specific CD8+ T cells in the tumor. Although SAHA alone appears to elicit CD8+ T cell immune responses in the tumor, only the combination treatment generated the best antitumor outcomes (Figure 1B). Bortezomib have been shown to enhance the immunogenicity in the tumor microenvironment. Importantly, our combination treatment acts on both antigen-specific CD8+ T cells and tumor cells. The synergistic interaction between the potent CD8+ T cell immune activation following SAHA treatment and the immunostimulating effects of bortezomib likely contributed to the observed potent therapeutic effect. It will be of great interest to elucidate the precise mechanisms of the synergistic therapeutic interactions, which warrants further investigation.

We observed significant enhancement of tumor-specific immunity following treatment with bortezomib and SAHA. Our observations suggest that the combination treatment enhances release of tumor antigen from tumor cells, which are subsequently processed and presented by professional antigen-presenting cells (APCs). The APCs then prime antigen-specific CD8+ T cells through a cross-priming mechanism (for review see [26]). Although here we only characterized E7-specific CD8+ T cells (Figure 2), other tumor antigens were likely released (E6, for example) promoting tumor-specific immunity. It is now clear the tumor-specific immunity can contribute to antitumor effects. Thus, the tumor-specific immunity generated through the combination treatment of bortezomib and SAHA may contribute to the therapeutic antitumor effect in addition to the direct killing of tumor cells by these chemotherapeutic drugs.

We found that tumors treated with both bortezomib and SAHA resulted in a more potent activation of antigen-specific CD8+ T cell immune responses (Figure 4). This could be the result of enhanced antigen processing or presentation through the MHC class I molecule following the combination treatment. It will be helpful to understand the precise mechanisms for such enhanced activation and should be investigated in future studies. Interestingly, our data also show that the combination treatment rendered tumor cells more susceptible to antigen-specific CD8+ T cell killing (Figure 5). Taken together, these data suggest that treatment of tumor-bearing mice with bortezomib and SAHA will potentially create potent immune-mediated therapeutic antitumor effects through not only the enhancement of tumor-specific immunity, but also the enhanced susceptibility of the tumor cells to antigen-specific CD8+ T cell-mediated killing.

Previously, our lab has used bortezomib with therapeutic HPV DNA vaccine for the control of TC-1 tumors [27]. We found that the combination of CRT/E7 DNA vaccine and bortezomib generated more potent antitumor effects in TC-1 tumor-bearing mice compared to either therapy alone. Considering these previous observations as well as our current ones, we suspect that the CRT/E7 DNA vaccine could be combined with bortezomib and SAHA in a highly potent therapy for cervical cancer.

## Conclusions

Taken together, our data suggest that the host immune response elicited by the treatment of HPV-associated tumors with both bortezomib and SAHA represents an important pathway contributing to the observed antitumor effects. Both SAHA and bortezomib are commercially available for the treatment of cutaneous T cell lymphoma and multiple myeloma/mantle cell lymphoma, respectively. In addition, the drug combination has been tested in some clinical trials in patients with advanced cancer. Furthermore, the combination of bortezomib and SAHA has been shown to generate synergistic killing of cervical cancer cell lines directly [12]. Thus, the current study serves an important foundation for the future clinical application of both drugs for the treatment of cervical cancer.

## Additional files

Additional file 1: Figure S1. Half maximal inhibitory concentration (IC50) of bortezomib and SAHA in TC-1 tumor cells. To determine the viability of TC-1 cells after bortezomib and SAHA treatment, 3-(4,5-dimethylthiazol-2-yl)-2,5-diphenyl-tetrazolium bromide (MTT, 5 mg/ml) assay was performed. TC-1 cells were plated in 96-well plates at a density of 1 × 10^3^ cells/well and incubated at 37°C in the presence of 5% CO_2_ for 12 hours. The cells were then treated with various concentrations of bortezomib or SAHA for 48 h. At the end of the treatment period, MTS reagent was added to each well, and the plate was incubated for 4 h at 37°C in the dark. After incubation, the absorbance was measured at 490 nm using the VERSA Max Microplate Reader. Data from three independent experiments were analyzed and normalized to the absorbance of wells containing media only (0%) and untreated cells (100%). The IC50 values were calculated from sigmoidal dose-response curves using MS Excel software. A. Line graph depicting the IC50 of bortezomib in the TC-1 tumor cell line. B. Line graph depicting the IC50 of SAHA in the TC-1 tumor cell line. Additional file 2: Figure S2. Images and weight of TC-1 tumors after treatment. Groups of female C57BL/6 mice were injected with 3 × 10^4^ of TC-1 tumor cells subcutaneously. Five days after tumor cell injection, the mice were treated through intraperitoneal injection with one of the following regimens: vehicle, bortezomib alone (1 mg/kg, once every 3 days), SAHA alone (30 mg/kg, once per day), or the combination of bortezomib and SAHA as described in Figure 1A. 4 days after last treatment, the tumors were resected from the mice and the weight of tumor was measured. A. Images of TC-1 tumors. B. Summary of the weight of subcutaneous TC-1 tumor.

## Abbreviations

HDAC: Histone deacetylase
HDACi: Histone deacetylase inhibitor
SAHA: Suberoylanilide hydroxamic acid
MHC: Major histocompatibility complex
PBMC: Peripheral blood mononuclear cell
SE: Standard error
CRT: Calreticulin
APC: Antigen-presenting cell
