# Supplementary figures and images for: Combination of proteasome and HDAC inhibitor enhances HPV16 E7-specific CD8+ T cell immune response and antitumor effects in a preclinical cervical cancer model
Source: J Biomed Sci. 2015 Jan 16;22(1):7. doi: 10.1186/s12929-014-0111-1 (PMC4298946; doi:10.1186/s12929-014-0111-1)

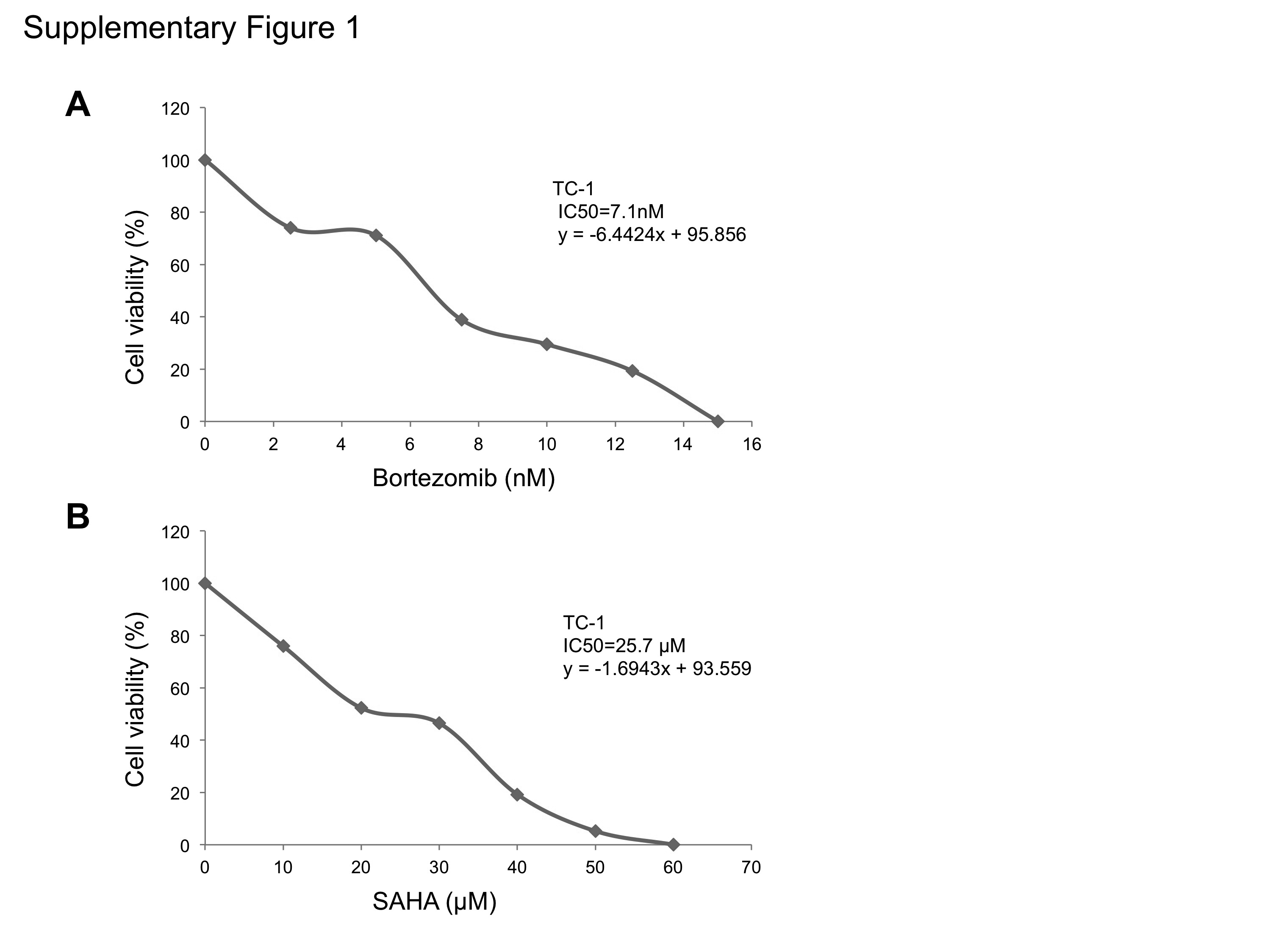

Supplement: Additional file 1: Figure S1. — Half maximal inhibitory concentration (IC50) of bortezomib and SAHA in TC-1 tumor cells. To determine the viability of TC-1 cells after bortezomib and SAHA treatment, 3-(4,5-dimethylthiazol-2-yl)-2,5-diphenyl-tetrazolium bromide (MTT, 5 mg/ml) assay was performed. TC-1 cells were plated in 96-well plates at a density of 1 × 10^3^ cells/well and incubated at 37°C in the presence of 5% CO2 for 12 hours. The cells were then treated with various concentrations of bortezomib or SAHA for 48 h. At the end of the treatment period, MTS reagent was added to each well, and the plate was incubated for 4 h at 37°C in the dark. After incubation, the absorbance was measured at 490 nm using the VERSA Max Microplate Reader. Data from three independent experiments were analyzed and normalized to the absorbance of wells containing media only (0%) and untreated cells (100%). The IC50 values were calculated from sigmoidal dose-response curves using MS Excel software. A. Line graph depicting the IC50 of bortezomib in the TC-1 tumor cell line. B. Line graph depicting the IC50 of SAHA in the TC-1 tumor cell line. [file 12929_2014_111_MOESM1_ESM.tiff]

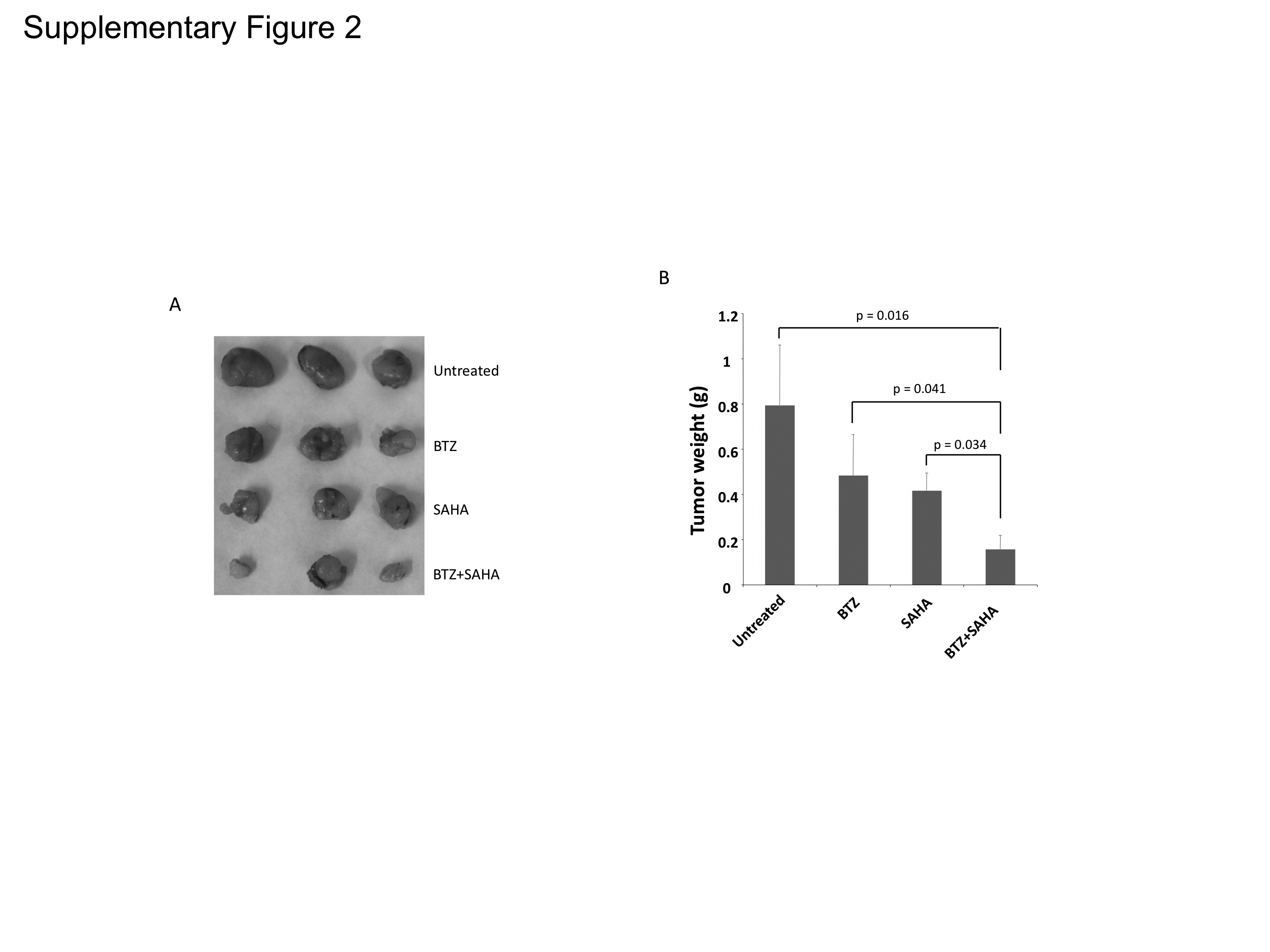

Supplement: Additional file 2: Figure S2. — Images and weight of TC-1 tumors after treatment. Groups of female C57BL/6 mice were injected with 3 × 10^4^ of TC-1 tumor cells subcutaneously. Five days after tumor cell injection, the mice were treated through intraperitoneal injection with one of the following regimens: vehicle, bortezomib alone (1 mg/kg, once every 3 days), SAHA alone (30 mg/kg, once per day), or the combination of bortezomib and SAHA as described in Figure 1A. 4 days after last treatment, the tumors were resected from the mice and the weight of tumor was measured. A. Images of TC-1 tumors. B. Summary of the weight of subcutaneous TC-1 tumor. [file 12929_2014_111_MOESM2_ESM.tiff]
